# A *Drosophila *protein-interaction map centered on cell-cycle regulators

**DOI:** 10.1186/gb-2004-5-12-r96

**Published:** 2004-11-26

**Authors:** Clement A Stanyon, Guozhen Liu, Bernardo A Mangiola, Nishi Patel, Loic Giot, Bing Kuang, Huamei Zhang, Jinhui Zhong, Russell L Finley

**Affiliations:** 1 Center for Molecular Medicine & Genetics, Wayne State University School of Medicine, 540 E. Canfield Avenue, Detroit, MI 48201, USA; 2 CuraGen Corporation, 555 Long Warf Drive, New Haven, CT 06511, USA; 3 Department of Biochemistry and Molecular Biology, Wayne State University School of Medicine, 540 E. Canfield Avenue, Detroit, MI 48201, USA

## Abstract

**Background:**

Maps depicting binary interactions between proteins can be powerful starting points for understanding biological systems. A proven technology for generating such maps is high-throughput yeast two-hybrid screening. In the most extensive screen to date, a Gal4-based two-hybrid system was used recently to detect over 20,000 interactions among *Drosophila *proteins. Although these data are a valuable resource for insights into protein networks, they cover only a fraction of the expected number of interactions.

**Results:**

To complement the Gal4-based interaction data, we used the same set of *Drosophila *open reading frames to construct arrays for a LexA-based two-hybrid system. We screened the arrays using a novel pooled mating approach, initially focusing on proteins related to cell-cycle regulators. We detected 1,814 reproducible interactions among 488 proteins. The map includes a large number of novel interactions with potential biological significance. Informative regions of the map could be highlighted by searching for paralogous interactions and by clustering proteins on the basis of their interaction profiles. Surprisingly, only 28 interactions were found in common between the LexA- and Gal4-based screens, even though they had similar rates of true positives.

**Conclusions:**

The substantial number of new interactions discovered here supports the conclusion that previous interaction mapping studies were far from complete and that many more interactions remain to be found. Our results indicate that different two-hybrid systems and screening approaches applied to the same proteome can generate more comprehensive datasets with more cross-validated interactions. The cell-cycle map provides a guide for further defining important regulatory networks in *Drosophila *and other organisms.

## Abstract 2

A *Drosophila *protein-protein interaction map was constructed using the LexA system, complementing a previous map using the GAL4 system and adding many new interactions.

## Background

Protein-protein interactions have an essential role in a wide variety of biological processes. A wealth of data has emerged to show that most proteins function within networks of interacting proteins, and that many of these networks have been conserved throughout evolution. Although some of these networks constitute stable multi-protein complexes while others are more dynamic, they are all built from specific binary interactions between individual proteins. Maps depicting the possible binary interactions among proteins can therefore provide clues not only about the functions of individual proteins but also about the structure and function of entire protein networks and biological systems.

One of the most powerful technologies used in recent years for mapping binary protein interactions is the yeast two-hybrid system [1]. In a yeast two-hybrid assay, the two proteins to be tested for interaction are expressed with amino-terminal fusion moieties in the yeast *Saccharomyces cerevisiae*. One protein is fused to a DNA-binding domain (BD) and the other is fused to a transcription activation domain (AD). An interaction between the two proteins results in activation of reporter genes that have upstream binding sites for the BD. To map interactions among large sets of proteins, the BD and AD expression vectors are placed initially into different haploid yeast strains of opposite mating types. Pairs of BD and AD fused proteins can then be tested for interaction by mating the appropriate pair of yeast strains and assaying reporter activity in the resulting diploid cells [2]. Large arrays of AD and BD strains representing, for example, most of the proteins encoded by a genome, have been constructed and used to systematically detect binary interactions [3-6]. Most large-scale screens have used such arrays in a library-screening approach in which the BD strains are individually mated with a library containing all of the AD strains pooled together. After plating the diploids from each mating onto medium that selects for expression of the reporters, the specific interacting AD-fused proteins are determined by obtaining a sequence tag from the AD vector in each colony.

High-throughput two-hybrid screens have been used to map interactions among proteins from bacteria, viruses, yeast, and most recently, *Caenorhabditis elegans *and *Drosophila melanogaster *[4-10]. Analyses of the interaction maps generated from these screens have shown that they are useful for predicting protein function and for elaborating biological pathways, but the analyses have also revealed several shortcomings in the data [11-13]. One problem is that the interaction maps include many false positives - interactions that do not occur *in vivo*. Unfortunately, this is a common feature of all high-throughput methods for generating interaction data, including affinity purification of protein complexes and computational methods to predict protein interactions [11-14]. A solution to this problem has been suggested by several studies that have shown that the interactions detected by two or more different high-throughput methods are significantly enriched for true positives relative to those detected by only one approach [11-13]. Thus it has become clear that the most useful protein-interaction maps will be those derived from combinations of cross-validating datasets.

A second shortcoming of the large-scale screens has been the high rate of false negatives, or missed interactions. This is evident from comparing the high-throughput data with reference data collected from published low-throughout studies. Such comparisons with two-hybrid maps from yeast [13] and *C. elegans *[5], for example, have shown that the high-throughput data rarely covers more than 13% of the reference data, implying that only about 13% of all interactions are being detected. The finding that different large datasets show very little overlap, despite having similar rates of true positives, supports the conclusion that high-throughput screens are far from saturating [10,12]. For example, three separate screening strategies were used to detect hundreds of interactions among the approximately 6,000 yeast proteins, and yet only six interactions were found in all three screens [10]. These results suggest that many more interactions might be detected simply by performing additional screening, or by applying different screening strategies to the same proteins. In addition, anecdotal evidence has suggested that the use of two-hybrid systems based on different fusion moieties may broaden the types of protein interactions that can be detected. In one study, for example, screens performed using the same proteins fused to either the LexA BD or the Gal4 BD produced only partially overlapping results, and each system detected biologically significant interactions missed by the other [15]. Thus, the application of different two-hybrid systems and different screening strategies to a proteome would be expected to provide more comprehensive datasets than would any single screen.

We set out to map interactions among the approximately 14,000 predicted *Drosophila *proteins by using two different yeast two-hybrid systems (LexA- and Gal4-based) and different screening strategies. Results from the screens using the Gal4 system have already been published [6]. In that study, Giot *et al*. successfully amplified 12,278 *Drosophila *open reading frames (ORFs) and subcloned a majority of them into the Gal4 BD and Gal4 AD expression vectors by recombination in yeast. They screened the arrays using a library-screening approach and detected 20,405 interactions involving 7,048 proteins. To extend these results we subcloned the same amplified *Drosophila *ORFs into vectors for use in the LexA-based two-hybrid system, and constructed arrays of BD and AD yeast strains for high-throughput screening. Our expectation was that maps generated with these arrays would include interactions missed in previous screens, and would also partially overlap the Gal4 map, providing opportunities for cross-validation.

Initially, we screened for interactions involving proteins that are primarily known or suspected to be cell-cycle regulators. We chose cell-cycle proteins as a starting point for our interaction map because cell-cycle regulatory systems are known to be highly conserved in eukaryotes, and because previous results have suggested that the cell-cycle regulatory network is centrally located within larger cellular networks [16]. This is most evident from examination of the large interaction maps that have been generated for yeast proteins using yeast two-hybrid and other methods. Within these maps there are more interactions between proteins that are annotated with the same function (for example, 'Pol II transcription', 'cell polarity', 'cell-cycle control') than between proteins with different functions, as expected for a map depicting actual functional connections between proteins. Interestingly, however, certain functional groups have more inter-function interactions than others. Proteins annotated as 'cell-cycle control', in particular, were frequently connected to proteins from a wide range of other functional groups, suggesting that the process of cell-cycle control is integrated with many other cellular processes [16]. Thus, we set out to further elaborate the cell-cycle regulatory network by identifying new proteins that may belong to it, and new connections to other cellular networks.

## Results

### Construction of an extensive protein interaction map centered on cell-cycle regulators by high-throughput two-hybrid screening

We used the same set of 12,278 amplified *Drosophila *full-length ORFs from the Gal4 project [6] to generate yeast arrays for use in a modified LexA-based two-hybrid system (see Materials and methods). In the LexA system the BD is LexA and the AD is B42, an 89-amino-acid domain from *Escherichia coli *that fortuitously activates transcription in yeast [17]. In the version that we used, both fusion moieties are expressed from promoters that are repressed in glucose so that their expression can be repressed during construction and amplification of the arrays [18]. Previous results have shown that this prevents the loss of genes encoding proteins that are toxic to yeast, and that interactions involving such proteins can be detected by inducing their expression only on the final indicator media [18,19]. The ORFs were subcloned into the two vectors by recombination in yeast as previously described [3,6], and the yeast transformants were arrayed in a 96-well format. The resulting BD and AD arrays each have approximately 12,000 yeast strains, over 85% of which have a full-length *Drosophila *ORF insert (see Materials and methods). For all strains involved in an interaction reported here, the plasmid was isolated and the insert was sequenced to verify the identity of the ORF.

As a first step toward generating a LexA-based protein-interaction map, we chose 152 BD-fused proteins that were either known or homologous to regulators of the cell cycle or DNA damage repair (see Additional data file 2). We used all 152 proteins as 'baits' to screen the 12,000-member AD array. We used a pooled mating approach [19] in which individual BD bait strains are first mated with pools of 96 AD strains. For pools that are positive with a particular BD, the corresponding 96 AD strains are then mated with that BD in an array format to identify the particular interacting AD protein(s). We had previously shown that this approach is very sensitive and allows detection of interactions involving proteins that are toxic to yeast or BD fused proteins that activate transcription on their own [19]. Moreover, the final assay in this approach is a highly reproducible one-on-one assay between an AD and a BD strain, in which the reporter gene activities are recorded to provide a semi-quantitative measure of the interaction. Using this approach we detected 1,641 reproducible interactions involving 93 of the bait proteins. We also performed library screening [6] with a subset of the 152 baits that did not activate the reporter genes on their own. This resulted in the detection of 173 additional interactions with 57 bait proteins. Thirty-nine interactions were found by both approaches, and these involved 21 of the 44 BD genes active in both approaches. There were 95 BD genes for which interaction data was obtained by the pooled mating approach, and 59 active BD genes in the library screening approach. The average number of interactions was 18 per BD gene in the pooled mating data, while the library screening data had an average of only four interactions per active BD gene. The average level of reporter activation for the 39 interactions that were detected in both screens was significantly higher than the average of all interactions (see Additional data file 3), suggesting that the weaker interactions are more likely to be missed by one screen or another, even though they are reproducible once detected.

Altogether we detected interactions with 106 of the 152 baits, which resulted in a protein-interaction map with 1,814 unique interactions among the products of 488 genes (see Additional data file 3). The map includes interactions that were already known or that could be predicted from known orthologous or paralogous interactions (see below). The map also includes a large number of novel interactions, including many involving functionally unclassified proteins.

### Evaluation of the LexA-based protein interaction map

**Figure 1:**
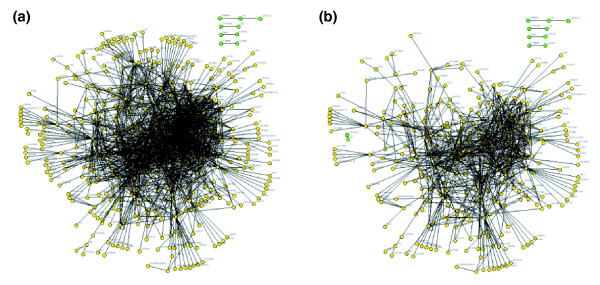
A protein interaction map centered on cell cycle regulators. **(a) **The entire map includes 1,814 unique interactions (lines) among the proteins encoded by 488 genes (circles). The map has five distinct networks; one network contains 479 (98%) of the proteins, one has three proteins, and three have two proteins (upper right, green circles). **(b) **The interconnectedness of the map does not depend strongly on the proteins with the most interactions. The map shown comprises data filtered to remove proteins with more than 30 interactions (*k *> 30), leaving 792 interactions among 343 proteins. This produced only one additional network, which has two proteins (green circles on the left of (b)); 97% of the proteins still belong to a single large network. Further deletion of proteins with *k *> 20 removes an additional 469 interactions, which creates only four additional small networks and leaves 85% of the proteins in a single network (data not shown). A high-resolution version of this figure with live links to gene information can be drawn using a program available at [47].

As is common with data derived from high-throughput screens, the number of novel interactions detected was large, making direct *in vivo *experimental verification impracticable. Thus, we set out to assess the quality of the data by examining the topology of the interaction map, by looking for enrichment of genes with certain functions, and by comparing the LexA map with other datasets. First we examined the topology of the interaction map, because recent studies have shown that cellular protein networks have certain topological features that correlate with biological function [20]. In our interaction map, the number of interactions per protein (*k*) varies over a broad range (from 1 to 84) and the distribution of proteins with *k *interactions follows a power law, similar to previously described protein networks [6,21]. Most (98%) of the proteins in the map are linked together into a single network component by direct or indirect interactions (Figure 1a). The network has a small-world topology [22], characterized by a relatively short average distance between any two proteins (Table 1) and highly interconnected clusters of proteins. Removal of the most highly connected proteins from the map does not significantly fragment the network, indicating that the interconnectivity is not simply due to the most promiscuously interacting proteins (Figure 1b). In other interaction maps generated with randomly selected baits, proteins with related functions tend to be clustered into regions that are more highly interconnected than is typical for the map as a whole [5,6,16]. Moreover, interactions within more highly interconnected regions of a protein-interaction map tend to be enriched for true positives [6,23-25]. Thus, the overall topology of the interaction map that we generated is consistent with that of other protein networks, and in particular, with the expectation for a network enriched for functionally related proteins.

Next we assessed the list of proteins in the interaction map to look for enrichment of proteins or pairs of proteins with particular functions. An interaction map with a high rate of biologically relevant interactions should have a high frequency of interactions between pairs of proteins previously thought to be involved in the same biological process. Among the 488 proteins in the map, 153 have been annotated with a putative biological function using the Gene Ontology (GO) classification system [26,27]. Because we used a set of BD fusions enriched for cell-cycle and DNA metabolic functions, we expected to see similar enrichments in the list of interacting AD fusions, as well as more interactions between genes with these functions. Both of these expectations are borne out. In the list of BD genes, both cell-cycle and DNA metabolism functions are enriched approximately 17-fold compared to similarly sized lists of randomly selected proteins (*P *< 0.00002). In the AD list, these two functions are enriched four- and threefold, respectively (Table 2). The frequency with which interactions occur between pairs of proteins annotated for DNA metabolism is five times more than expected by chance; similarly, cell-cycle genes interact with each other six times more frequently than expected (*P *< 0.001). Thus, the enrichment for proteins and pairs of interacting proteins annotated with the same function suggests that many of the novel interactions will be biologically significant. It also suggests that the map will be useful for predicting the functions of novel proteins on the basis of their connections with proteins having known functions, as described for other interaction maps [16,28].

### Comparison of the *Drosophila *protein-interaction maps

Direct comparison of the LexA cell-cycle map with the Gal4 data revealed that only 28 interactions were found in common between the two screens (Table 1). Moreover, more than a quarter of the proteins in the LexA map were absent from the Gal4 proteome-wide map. Among the 106 baits that had interactions in the LexA map, for example, 60 failed to yield interactions in the Gal4 proteome-wide map, even though all but six of these were successfully cloned in the Gal4 arrays [6] (see Additional data file 6). Similarly, 46 of the 152 LexA baits that we used failed to yield interactions from our work, yet 14 of these had interactions in the Gal4 map. Thus, the lack of overlap between the two datasets is partly due to their unique abilities to detect interactions with specific proteins. Nevertheless, for the 347 proteins common to both maps, the two screens combined to detect 1428 interactions, and yet only 28 of these were in both datasets. This indicates that the two screens detected mostly unique interactions even among the same set of proteins. Comparison with a set of approximately 2,000 interactions recently generated in an independent two-hybrid screen [29] showed only three interactions in common with our data, in part because only eight of the same bait proteins were used successfully in both screens.

Although only 28 interactions were found in both the Gal4 map and our map, this rate of overlap is significantly greater than expected by chance (*p *< 10^-6^; Table 1). To show this, we generated 10^6 ^random networks having the same BD proteins, total interactions and topology as the LexA map, and found that none of these random maps shared more than two interactions in common with the Gal4 map. To assess the relative quality of the 28 common interactions we used the confidence scores assigned to them by Giot *et al*. [6]. They used a statistical model to assign confidence scores (from 0 to 1), such that interactions with higher scores are more likely to be biologically relevant than those with lower scores. The average confidence scores of the 28 interactions in common with our LexA data (0.63), was higher than the average for all 20,439 Gal4 interactions (0.34), or for random samplings of 28 Gal4 interactions (0.32; *P *< 0.0001), indicating that the overlap of the two datasets is significantly enriched for biologically relevant interactions. Thus, the detection of interactions by both systems could be used as an additional measure of reliability. The surprisingly small number of common interactions, however, severely limits the opportunities for cross-validation, and suggests that both datasets are far from comprehensive.

An alternative explanation for the small proportion of common interactions is the possible presence of a large number of false positives in one or both datasets. The estimation of false-positive rates is challenging, in part because it is difficult to prove that an interaction does not occur under all *in vivo *conditions, and also because the number of potential false positives is enormous. Nevertheless, the relative rates of false positives between two datasets can be inferred by comparing their estimated rates of true positives [11-13]. To compare true-positive rates between the LexA and Gal4 datasets, we looked for their overlap with several datasets that are thought to be enriched for biologically relevant interactions (Table 3). These include a reference set of published interactions involving the proteins that were used as baits in both the LexA and Gal4 screens; interactions between the *Drosophila *orthologs of interacting yeast or worm proteins (orthologous interactions or 'interlogs' [30,31]); and between proteins encoded by genes known to interact genetically, which are more likely to physically interact than random pairs of proteins [32,33]. As expected, the overlap with these datasets is enriched for higher confidence interactions. The average confidence scores for the Gal4 interactions in common with the yeast interlogs, worm interlogs and *Drosophila *genetic interactions are 0.63, 0.68 and 0.80, respectively, substantially higher than the average confidence scores for all Gal4 interactions (0.34). This supports the notion that these datasets are enriched for true-positive interactions relative to randomly selected pairs of proteins. We found that the fractions of LexA- and Gal4-derived interactions that overlap with these datasets are similar (Table 3). For example, 25 (1.4%) of the 1814 LexA interactions and 294 (1.4%) of the 20,439 Gal4 interactions have yeast interlogs. This suggests that the LexA and Gal4 two-hybrid datasets have similar percentages of true positives, and thus similar rates of false positives. They also appear to have similar rates of false negatives, which may be over 80% if calculation is based on the lack of overlap with published interactions (Table 3). This supports the explanation that the main reason for the lack of overlap between the datasets is that neither is a comprehensive representation of the interactome, and suggests that a large number of interactions remain to be detected.

### Biologically informative interactions

**Figure 2:**
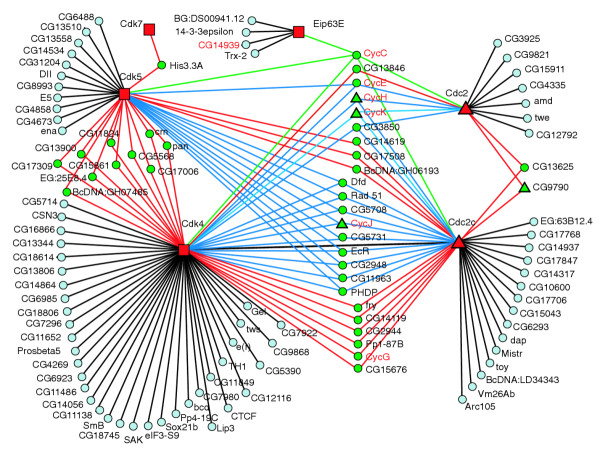
A map of the interactions involving cyclin-dependent kinases (Cdks). All the interactions involving at least one of the six Cdks (Cdc2, Cdc2c, Cdk4, Cdk5, Cdk7) and Eip63E (red nodes) are shown. All the Cdks except Cdk7 interacted with at least two cyclins (red text). All the cyclins interacted with at least two Cdks, with the exception of the novel cyclin-like protein CG14939, which only interacted with Eip63E. Other known or paralogous interactions include, Cdc2c-dap, Cdc2-twe, and the interactions of Cdc2 and Cdc2c with CG9790, a Cks1-like protein. Proteins are depicted according to whether they appear in the map only as BD fusions (squares), only as AD fusions (circles), or as both BD and AD fusions (triangles). Proteins connected to more than one Cdk are green. Interactions are colored if they involve proteins contacting two Cdks (red), three Cdks (blue), or five Cdks (green).

Further inspection of the LexA cell-cycle interaction map revealed biologically informative interactions and additional insights for interpreting high-throughput two-hybrid data. For example, we expected to observe interactions between cyclins and cyclin-dependent kinases (Cdks), which have been shown to interact by a number of assays. Our interaction map includes six proteins having greater than 40% sequence identity to Cdk1 (also known as Cdc2). A map of all the interactions involving these proteins reveals that they are multiply connected with several cyclins (Figure 2). For example, all of the known cyclins in the map interacted with at least two of the Cdk family members. The map includes 20 interactions between five Cdks and six known cyclins plus one uncharacterized protein, CG14939, which has sequence similarity to cyclins. Only one of these interactions (Cdc2c-CycJ) is known to occur *in vivo *[34], and several others are thought not to occur *in vivo *(for example Cdc2-CycE [35]). Similarly, the Gal4 interaction map has three Cdk-cyclin interactions [6], including one known to occur *in vivo *(Cdk4-CycD) and two that do not occur *in vivo *[35].

Thus, while some of these interactions are false positives in the strictest sense, the data is informative nevertheless, as it clearly demonstrates a high incidence of paralogous interactions - where pairs of interacting proteins each have paralogs, some combinations of which also interact *in vivo*. Such patterns are consistent with potential interactions between members of different protein families, even though they do not reveal the precise pair of proteins that interact *in vivo*. This class of informative false positives may be common in two-hybrid data where the interaction is assayed out of biological context. Experimentally reproducible interactions, whether or not they occur *in vivo*, can be used to discover interacting protein motifs or domains [6,36]. They can also suggest functional relationships between protein families and guide experiments to establish the actual *in vivo *interactions and functions of specific pairs of interacting proteins.

**Figure 3:**
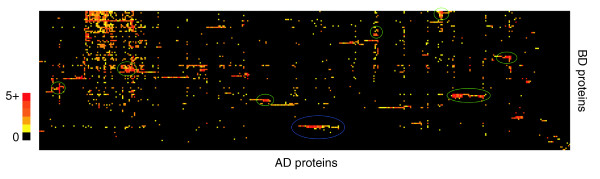
Proteins clustered by their interaction profiles. BD fused proteins (*y*-axis) and AD fused proteins (*x*-axis) were independently clustered according to the similarities of their interaction profiles using a hierarchical clustering algorithm (see Materials and methods). An interaction between a BD and AD protein is indicated by a small colored square. The squares are colored according to the level of two-hybrid reporter activity, which is the sum of LEU2 (0-3) and lacZ (0-5) scores, where higher scores indicate more reporter activity (1, yellow; 5+, red). The cluster circled in blue (center) corresponds to interactions involving SkpA, SkpB and SkpC BD fusions, which are mapped in Figure 4. Maps of other clusters (circled in green) are shown in Additional data file 7. The large cluster at upper left is due primarily to AD proteins that interact with many different BD proteins. A larger version of the figure with the gene names indicated in the axes is in Additional data file 8.

**Figure 4:**
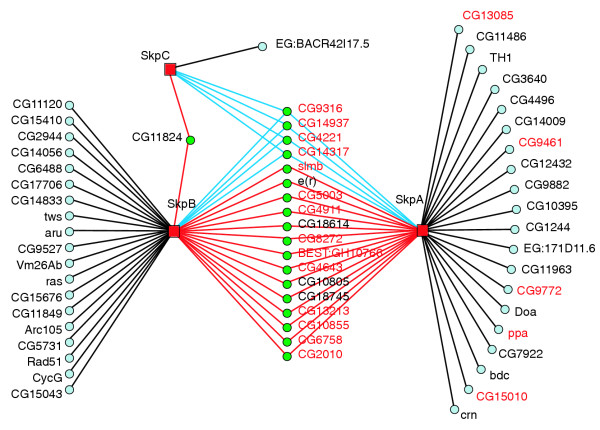
A map of the interactions in the Skp cluster. All the interactions with the BD fusions SkpA, SkpB and SkpC, are shown. Proteins (green) interacting with more that one Skp paralog are enriched for proteins possessing an F-box domain (red text). Other colors and shapes are as in Figure 2.

The Cdk subgraph also illustrates that proteins with similar interaction profiles may have related functions or structural features. To look for other groups of proteins having similar interaction profiles we used a hierarchical clustering algorithm to cluster BD and AD fusion proteins according to their interactions (see Materials and methods). The resulting clustergram reveals several groups of proteins with similar interaction profiles (Figure 3). One of the most prominent clusters (Figure 3, circled in blue) includes three related proteins involved in ubiquitin-mediated proteolysis, SkpA, SkpB and SkpC. Skp proteins are known to interact with F-box proteins, which act as adaptors between ubiquitin ligases, known as SCF (Skp-Cullin-F-box) complexes, and proteins to be targeted for destruction by ubiquitin-mediated proteolysis [37]. A map of the interactions involving the Skp proteins shows a group of 21 AD proteins that each interact with two or three of the Skp proteins (Figure 4). This group is highly enriched for F-box proteins, including 13 of the 15 F-box proteins in the AD list; the other two F-box proteins interacted with only one Skp (Figure 4). Several of the interactions in common with the Gal4 data are also in the Skp cluster, and 12 out of 16 of these involve proteins that interact with two or more Skp proteins.

Thus, the Skp cluster provides another example of how proteins with similar interaction profiles may be structurally or functionally related, and how such clusters may be enriched for biologically relevant interactions. This is consistent with previous results showing that protein pairs often have related functions if they have a significantly larger number of common interacting partners than expected by chance [24,38]. These groups of proteins are likely to be part of more extensive functional clusters that could be identified by more sophisticated topological analyses (for example [39-44]. Maps showing several other major clusters derived from the cluster-gram are shown in Additional data file 7.

The interaction profile data is statistically confirmed by domain-pairing data, which shows that certain pairs of domains are found within interacting pairs of proteins more frequently than expected by chance (Table 4). These include the Skp domain and F-box pair, the protein kinase and cyclin domains, and several less obvious pairings. For example, the cyclin and kinase domains are observed to be associated with various zinc-finger and homeodomain proteins, and the kinase domain with a number of nucleic-acid metabolism domains (Table 4). A similar analysis of the Gal4 data, performed by Giot *et al*. [6], revealed a number of significant domain pairings, including the Skp/F-box and the kinase/cyclin pairs and several others found in the LexA dataset. Therefore, although the number of proteins in the LexA dataset is relatively small, domain associations are observed in the data, demonstrating that a high-density interaction map, with a high average number of interactions per protein, provides insight into patterns of domain interactions that is equally valuable as that obtained from a proteome-wide map.

## Discussion

Proteome-wide maps depicting the binary interactions among proteins provide starting points for understanding protein function, the structure and function of protein complexes, and for mapping biological pathways and regulatory networks. High-throughput approaches have begun to generate large protein-interaction maps that have proved useful for functional studies, but are also often plagued by high rates of false positives and false negatives. Several analyses have shown that the set of interactions detected by more than one high-throughout approach is enriched for biologically relevant interactions, suggesting that the application of multiple screens to the same set of proteins results in higher-confidence, cross-validated interactions [11-13]. Such cross-validation has been limited, however, by the lack of overlap among high-throughput datasets. Here we describe initial efforts to complement a recently published *Drosophila *protein interaction map that was generated using the Gal4 yeast two-hybrid system [6]. We constructed yeast arrays for use in the LexA-based two-hybrid system by subcloning approximately 12,000 *Drosophila *ORFs, using the same PCR amplification products used in the Gal4 project, into the LexA two-hybrid vectors. Initially, we used a novel pooled mating approach [19] to screen one of the 12,000-member arrays with 152 bait proteins related to cell cycle regulators. By using both a different screening approach and a different two-hybrid system, we expected to increase coverage and to validate some of the interactions detected by the Gal4 screens.

The level of coverage for a high-throughput screen can be estimated by determining the percentage of a reference dataset that was detected; reference sets have been derived from published low-throughput experiments, for example, which are considered to have relatively low false-positive rates. High-throughput two-hybrid data for yeast and *C. elegans *proteins were shown to cover only about 10-13% of the corresponding reference datasets [5,10,13]. Two factors may contribute to this lack of coverage. First, some interactions cannot be detected using the yeast two-hybrid system, even though they could be detected in low-throughput studies using other methods. Examples include interactions that depend on certain post-translational modifications, that require a free amino terminus or that involve membrane proteins. Second, high-throughput yeast two-hybrid screens often fail to test all possible combinations of interactions; in other words, the screens are not saturating or complete.

Although the relative contribution of these two factors is difficult to estimate, results from screens to map interactions among yeast proteins suggest that the major reason for the lack of coverage is that the screens are incomplete. Complete screens would identify all interactions that could possibly be detected by a given method; ideally therefore, two complete screens using the same method would identify all the same interactions. However, the rate of overlap among the different yeast proteome screens is low, even though they used very similar two-hybrid systems. Moreover, the overlap between screens is not significantly greater than the rate at which they overlap any reference set [4,10]. This is true even when only higher-confidence interactions are considered; for example, two large interaction screens of yeast proteins detected 39% and 65% of a higher-confidence dataset, respectively, but only 11% of the reference set was detected by both screens [12]. These results indicate that the lack of coverage in high-throughput two-hybrid data is largely due to incomplete screening, and that significantly larger datasets than those currently available will be needed before different datasets can be used to cross-validate interactions.

The rates of coverage and completeness from our high-throughput two-hybrid screening with *Drosophila *proteins are consistent with those for the yeast proteins. We used the LexA system to detect 1,814 reproducible interactions to complement the 20,439 interactions previously detected in a proteome-wide screen using the Gal4 system [6]. The overlap between the LexA and Gal4 screens is less than 2% of each dataset, whereas their overlap with a reference set was 17% and 14%, respectively, and only 2% of the reference set was detected by both screens (Table 2). Taken together, these results suggest that, like the yeast interaction data, both *Drosophila *datasets are far from complete and that many more interactions could be detected by additional two-hybrid screening.

The actual number of interactions that might be detected by complete two-hybrid screening might be roughly estimated from the partially overlapping datasets, as was performed for accurate estimation of the number of genes in the human genome [45,46]. In this approach, the overlap of two subsets, given that one subset is a homogeneous random sample of the whole, is sufficient to estimate the size of the whole. To make such an estimate with high-throughput two-hybrid data, however, it is necessary to first filter out false positives, as they are mostly different for the two datasets, as suggested by the fact that the nonoverlapping data has a lower rate of true positives than the overlapping data. Giot *et al*. estimated that at least 11% of the Gal4 interactions are likely to be biologically relevant, based on the prediction accuracy of their statistical model [6]. We found by comparison with other datasets that the rates of true positives are not substantially different between the LexA and Gal4 data (Table 3). Thus, if we use 11% as the minimal rate of true positives in each dataset, we obtain 200 true interactions from the LexA screen and 2,248 from the Gal4 screens. If we further assume that all of the 28 common interactions are true positives, we can estimate that complete screens should be able to detect around 16,000 true positive interactions (200 × 2,248/28). If each screening approach has a false-positive rate of 89%, then around 150,000 interactions from each approach would be required in order to create complete, cross-validating datasets, where the overlap would be comprised of true positives. This estimate is highly sensitive to both the frequency of true positives in the two datasets, and the number of positives in the overlap between the datasets; for example, if true-positive frequency is underestimated by only twofold, there will be four times as many interactions.

False-positive interactions have been classified as technical or biological [5]. A technical false positive is an artifact of the particular interaction assay, and the two proteins involved do not actually interact under any setting. A biological false positive is one in which the two proteins genuinely and reproducibly interact in a particular assay, but the interaction does not take place in a biological setting; for example, the interacting proteins may never be temporally or spatially co-localized *in vivo*. Using the approach described here, the interactions are shown to be reproducible during the one-on-one two-hybrid assays that are used to record reporter activity scores, suggesting that we have minimized the frequency of technical false positives.

We suggest that the biological false positives might be further classified as informative and non-informative. Informative false positives are interactions that do not occur *in vivo*, but that nevertheless have some biological basis for being detected and are potentially useful for guiding future experiments. In our data, for example, the Cdk and Skp proteins each interact with a different group of targets, which in turn interact with multiple Cdk or Skp proteins. From this data alone, we would accurately predict that Cdk proteins interact with cyclins, and that Skp proteins interact with F-box proteins, even though only some of the specific combinations are true *in vivo *partners. Similarly, from analysis of domain pairs in the LexA dataset, other patterns are evident, such as homeobox domains being associated with both protein kinase and cyclin domains (Table 4). Additional information or experimentation would be needed to determine which of the specific paralogous interactions function *in vivo*. Co-affinity purification, for example, might be used to directly test all possible pairs of paralogous interactions implied by the two-hybrid map. Alternatively, the genes encoding each possible pair of proteins could be examined for correlated expression patterns, for example, to suggest more likely pairs or to exclude pairs that are not coexpressed.

## Conclusions

We used high-throughput screening to detect 1,814 protein interactions involving many proteins with cell-cycle and related functions. The resulting interaction map is similar in quality to other large interaction maps and is predominated by previously unidentified interactions. The majority of the proteins in the map have not been assigned a biological function, and the map provides a first clue about the potential functions of these proteins by connecting them with characterized proteins or pathways. High-throughput interaction data such as this should allow researchers to quickly identify possible patterns of protein interactions for use in selecting additional functional assays to perform on their gene(s) of interest. This narrows down the number of potential assays necessary to establish function for a given gene from hundreds to just a handful; conversely, when studying a specific function, such as the cell cycle, interaction data can identify which few genes, selected from thousands, may have a role in the process. Just as the sequencing of various genomes has not allowed unambiguous ascription of biological function to the majority of the identified genes, mapping of an interactome by high-throughput methods does not allow final assignment of interaction capacity or of higher functionality to a protein. This requires additional experiments, guided by these and other high-throughput data. The results presented here show that extending and combining different two-hybrid datasets will allow further refinement of the selection of functional analyses to be performed for each protein of the proteome.

## Materials and methods

### Plasmids and strains

Yeast two-hybrid vectors used are related to those originally described for the LexA system [17]. The vector for expressing amino-terminal LexA DNA-binding domain (BD) fusions was pHZ5-NRT, which expresses fusions from the regulated *MAL62 *promoter [18]. The vector for expressing amino-terminal activation domain (AD) fusions from the *GAL1 *promoter was pJZ4-NRT, which was constructed from pJG4-5 [17] by replacing the *ADH1 *terminator with the *CYC1 *terminator and inserting the 5' and 3' recombination tags (5RT1 and 3RT1 [18]) into the cloning site downstream from the AD coding region. Construction details can be found in Additional data file 1. Maps and sequences are available at [47]. Yeast (*S. cerevisiae*) strain RFY231 (MAT *trp1*::*hisG his3 ura3-1 leu2*::3Lexop-*LEU2*) and RFY206 (Mat**a ***his3Δ200 leu2-3 lys2Δ201 ura3-52 trp1Δ*::*hisG*) were previously described [2,48]. RFY206 containing the *lacZ *reporter plasmid pSH18-34 [49] is referred to here as strain Y309.

### Yeast two-hybrid arrays

Two yeast arrays were constructed by homologous recombination (gap repair) in yeast [3]. We began with the 13,393 unique PCR products, which were generated using gene-specific primer pairs corresponding to the predicted *Drosophila *ORFs, from ATG to stop codon, described in Giot *et al*. [6]. For the AD array, we co-transformed RFY231 with each PCR product along with pJZ4-NRT that had been linearized with *Eco*RI and *Bam*HI, and selected recombinants on glucose minimal media lacking tryptophan. Five colonies from each transformation were picked and combined into a well of a 96-well plate. For the BD array, we co-transformed Y309 with each PCR product along with pHZ5-NRT that had been linearized with *Eco*RI and *Bam*HI, and selected recombinants on glucose minimal medium lacking histidine and uracil. BD clones used in the screens and AD clones showing positive interactions were sequenced to verify the ORF identities. See Additional data files for details.

### Two-hybrid screening

The BD fused proteins used as baits in our screens are listed in Additional data file 2. The AD array was screened using a two-phase pooled mating approach [19]. First, pools containing the 96 AD strains from each plate in the AD array were constructed by scraping strains grown on agar plates, dispersing in 15% glycerol, and aliquoting into a 96-well format; the 142 pools, representing approximately 13,000 AD strains, were arrayed on two 96-well plates. In the first phase, individual BD strains were mated with the 142 AD pools by dispensing 5-μl volumes of each culture onto YPD plates using a Biomek FX robot (Beckman Coulter). After 2 days growth at 30°C, yeast were replicated to medium selective for diploids, which have the AD, BD and *lacZ *reporter plasmids, and containing both galactose and maltose to induce expression of the AD and BD fusions, respectively. The plates also lacked leucine to assay for expression of the *LEU2 *reporter, and contained X-Gal (40 μg/ml) to assay for expression of *lacZ*. These plates were photographed after 5 days at 30°C and interactions were scored as described [19]. In the second phase of screening, single BD strains were mated with the appropriate panel(s) of 93 AD strains corresponding to the pools that were positive in the first phase. The *LEU2 *and *lacZ *reporters were assayed on separate plates: growth on plates lacking leucine was scored from 0 (no growth) to 3 (heavy growth); the extent of blue on the X-Gal plates was scored from 0 (white) to 5 (dark blue). After re-testing interactions (see Additional data files) the AD plasmids from interacting AD strains were rescued in bacteria and clones were sequenced to verify insert identity. Cloned plasmids were then reintroduced into RFY231 and used in all possible combinations of one-on-one mating operations with the appropriate BD strains to repeat the interaction assay a third time. The same set of BDs was also used to screen a pool of all approximately 13,000 AD strains using a library screening approach as described in the Additional data files. All interaction data from both screens are listed in Additional data file 3 and are also available at [47,50] and at IntAct [51] in the Proteomics Standards Initiative - Molecular Interactions (PSI-MI) standard exchange format [52].

### Data analysis

The interaction profiles for the BD fused proteins and AD fused proteins were independently clustered and are plotted in Figure 3 using Genespring software (Silicon Genetics). Protein-interaction map graphs in Figures 1, 2 and 4 and Additional data file 7 were drawn with a program developed by Lana Pacifico (L. Pacifico, F. Fotouhi and R.L.F., unpublished work) available at [47]. To determine *Drosophila *interlogs of yeast or worm interactions, a list of *Drosophila *proteins belonging to eukaryotic clusters of orthologous groups (KOGs) [53] was obtained from the National Center for Biotechnology Information (NCBI) [54]. Each fly protein was assigned one or more KOG IDs, based on the cluster(s) to which it belongs. A list of interactions among yeast (*S. cerevisiae*) proteins, derived mostly from high-throughput yeast two-hybrid screens [4,55] and from the determination of proteins in precipitated complexes [56,57], was obtained from the Comprehensive Yeast Genome Database [58,59].

For the interactions determined by precipitation of complexes, two lists were generated. One list includes the binary interactions between the bait protein and every protein that was co-precipitated, but not between the precipitated proteins (hub and spoke model). The second list included all possible binary interactions among the members of a complex (matrix model). The lists were each used to generate a list of interactions between KOG pairs, which in turn was used to generate a list of potential interactions between pairs of *Drosophila *proteins belonging to those KOGs. Similarly, *Drosophila*-worm (*C. elegans*) interlogs were determined using the list of interactions between worm proteins determined by high-throughput yeast two-hybrid screening [5]. *Drosophila *genetic interactions were obtained from Flybase [27,60]. To compare the two-hybrid data with other datasets we generated random interaction maps having the same BD proteins, total interactions and topological properties as the LexA or Gal4 data. The AD clones in each interaction list were indexed, an array of the same number of genes as the AD clones was randomly fetched from the *Drosophila *Release 3.1 genome [61] and these genes were used to replace the original AD clones at the same indexed positions.

Fifty thousand such random networks were generated for each two-hybrid dataset, and then compared with the yeast interlogs, worm interlogs, and genetic interactions to determine the amount of overlap expected by chance. *P *values represented the number of times that the observed number of overlapping interactions was detected in 50,000 iterations of a random network, divided by 50,000. In most cases *P *< 0.0002 (see Additional data file 6). Additional methods are in Additional data file 1. To compare the number of common interactions between the LexA and Gal4 maps with the number expected by chance, we generated 10^6 ^random LexA maps and found that they never contained more than two interactions in common with the Gal4 map; thus, the *P*-value for the 28 common interactions is significantly less than 10^-6^.

## Additional data files

The following additional data are available with the online version of this paper. Additional data file 1 contains Supplementary materials and methods; Additional data file 2 contains Supplementary Table 1, BD 'baits' used in the LexA screens; Additional data file 3 contains Supplementary Table 2, Interactions detected in the LexA screens; Additional data file 4 contains Supplementary Table 3, Enrichment of Gene Ontology classes, complete list; Additional data file 5 contains Supplementary Table 4, Enrichment of Domain pairs, complete list; Additional data file 6 contains Supplementary Table 5, *P*-values for overlap among datasets, and Supplementary Table 6, Interactions from the LexA and Gal4 screens that successfully used the same BD bait proteins; Additional data file 7 is a PDF containing Supplementary Figure 1, Interaction maps of other clusters; Additional data file 8 is a PDF containing Supplementary Figure 2, Proteins clustered by interaction profile; Additional data file 9 contains the legends to Supplementary Figures 1 and 2.

## Supplementary Material

Additional data file 1 Supplementary Materials and methods Click here for additional data file

Additional data file 2 Supplementary Table 1: BD 'baits' used in the LexA screens Click here for additional data file

Additional data file 3 Supplementary Table 2: Interactions detected in the LexA screens Click here for additional data file

Additional data file 4 Supplementary Table 3: Enrichment of Gene Ontology classes (the complete list) Click here for additional data file

Additional data file 5 Supplementary Table 4: Enrichment of Domain pairs (the complete list) Click here for additional data file

Additional data file 6 Supplementary Table 5: *P*-values for overlap among datasetsa nd Supplementary Table 6: Interactions from the LexA and Gal4 screens that successfully used the same BD bait proteins Click here for additional data file

Additional data file 7 Supplementary Figure 1: Interaction maps of other clusters Click here for additional data file

Additional data file 8 Supplementary Figure 2: Proteins clustered by interaction profile Click here for additional data file

Additional data file 9 The legends to Supplementary Figures 1 and 2 Click here for additional data file

## Figures and Tables

**Table 1:** Comparison of *Drosophila *protein-interaction maps generated by high-throughput yeast two-hybrid methods

	LexA cell-cycle map*	Gal4 proteome-wide map^†^	Common
Interactions	1,814	20,439	28
Proteins	488	6,951	347
Proteins as BD fusions	106	3,616	46
Proteins as AD fusions	403	5,425	250
Proteins as AD and BD	21	2,090	8
Degree exponent^‡^	1.72	1.91	NA
Mean path length^§^	3.3	4.1	NA

*The LexA interactions are from this study, listed in Additional data file 3. ^†^The Gal4 interactions are from Giot *et al*. [6]. The chance of observing more than two common interactions between the Gal4 map and a random network with the same topological properties as the LexA map is < 10^-6 ^(see Materials and methods). ^‡^The degree exponent and mean path length are topological properties of the networks. The degree exponent is γ in the equation P(*k*) = *k*^-γ^, where *k *is the degree or number of interactions per protein, and P(*k*) is the distribution of proteins with *k *interactions. ^§^The mean path length is the shortest number of links between a pair of proteins, averaged over all pairs in the network.

**Table 2:** Enrichment of the most frequently classified gene functions

Description	BD genes				AD Genes				Same-pair interactions			
			
	Exp	Rand	*P*	Ratio	Exp	Rand	*P*	Ratio	Exp	Rand	*P*	Ratio
Protein modification	30	2.92	<0.00002	10.3	21	11.12	0.00210	1.9	25	14.86	0.09916	1.7
**Cell cycle**	**22**	**1.27**	**<0.00002**	**17.3**	19	**4.83**	**<0.00002**	**3.9**	**26**	**4.40**	**0.00006**	**5.9**
**DNA metabolism**	**14**	**0.79**	**<0.00002**	**17.7**	**6**	**2.99**	**0.03006**	**2.0**	**6**	**1.15**	**0.00860**	**5.2**
Transcription	9	2.04	0.00002	4.4	14	7.77	0.01134	1.8	7	1.85	0.00242	3.8
Gametogenesis	9	1.49	<0.00002	6.0	13	5.69	0.00172	2.3	7	1.53	0.00072	4.6
Neurogenesis	8	1.91	0.00018	4.2	12	7.29	0.03142	1.6	14	3.75	0.00168	3.7
Cell-surface receptor-linked signal transduction	8	2.48	0.00088	3.2	11	9.39	0.23272	1.2	5	3.05	0.12498	1.6
**DNA repair**	**6**	**0.45**	**<0.00002**	**13.4**	**7**	**1.71**	**0.00030**	**4.1**	**3**	**0.28**	**0.00064**	**10.8**
Intracellular signaling cascade	6	0.65	0.00002	9.3	6	2.44	0.01036	2.5	3	0.98	0.03602	3.1
Imaginal disk development	5	0.80	0.00022	6.3	9	3.04	0.00092	3.0	3	0.45	0.00266	6.7
Average	11.7	1.48	0.00022	9.2	11.8	5.63	0.03209	2.4	9.9	3.23	0.02769	4.71

The top 10 most frequently classified BD gene functions, derived from GO biological process level 4 (see Materials and methods), are shown. The number of proteins or pairs of proteins in our experimental data (Exp) with each GO function is shown, alongside the average number of times the function would appear in a random interaction map (Rand) having the same topology and number of proteins (see Materials and methods), and the ratio of Exp/Rand. The functions listed are significantly enriched in the BD list, to *P *< 0.001, and most to *P *< 0.0003. Cell cycle, DNA metabolism and DNA repair (highlighted) are the three most proportionally enriched classifications in the BD list, These classes are also enriched for self-associations in the interaction list, with cell cycle and DNA metabolism around six- and fivefold enriched, while DNA repair is approximately 11-fold more self-associated than expected by chance. Of these three, DNA metabolism is not significantly enriched in the AD gene list (*P *> 0.03), while the other two classifications are approximately fourfold enriched. A complete list of all functions and function pairs found in the interaction data is in Additional data file 4.

**Table 3:** Overlap of two-hybrid data with datasets enriched for true positives

	Interactions	Overlap with LexA map (*N *= 1,814)	Overlap with Gal4 map (*N *= 20,439)	Overlap in common
Yeast interlogs (hub/spoke)*	67,238	23 (1.26%)	251 (1.23%)	4
Yeast interlogs (matrix)*	244,202	25 (1.38%)	294 (1.44%)	4
Worm interlogs*	37,863	3 (0.17%)	61 (0.30%)	0
Drosophila genetic^†^	2,751	4 (0.22%)	22 (0.11%)	1
Reference set^‡^	47	8 (0.44%)	6 (.03%)	2
Ref set (common BD)^§^	20	3 (0.17%)	2 (.01%)	0

*Yeast (*S. cerevisiae*) and worm (*C. elegans*) interlogs are predicted interactions between the *Drosophila *orthologs of interacting yeast and worm proteins; 'hub/spoke' and 'matrix' refer to the methods used to derive predicted binary interactions from the protein complex data (see Materials and methods). ^†^Genetic interactions were obtained from Flybase [27]. ^‡^The Reference set includes published interactions involving any of the 106 BD proteins in the LexA data. ^§^The subset of reference interactions involving proteins successfully used as BDs in both the Gal4 and LexA screens is also shown; no interactions from the reference set were found in both the LexA and Gal4 screens using the same BD baits. The chance of finding the indicated number of overlapping interactions with a random set of interactions was <10^-4 ^for all but the LexA overlaps with worm interlogs (*P *< 0.1436) or genetic interactions (*P *< 0.0024) (Additional data file 6).

**Table 4:** Domain pair enrichment

AD domain					BD domain					Domain pairings			
		
Name	Exp	Rand	Fold	*P*	Name	Exp	Rand	Fold	*P*	Exp	Rand	Fold	*P*
Cyclin	8	0.5	16	<0.00002	Protein kinase	30	1.7	18	<0.00002	38	0.6	60	<0.00002
F-box	17	1.2	15	<0.00002	Skp1	4	0.1	75	<0.00002	34	0.3	123	<0.00002
F-box	17	1.2	15	<0.00002	Skp1_POZ	4	0.1	65	<0.00002	34	0.3	123	<0.00002
Homeobox	9	2.9	3	0.00080	Protein kinase	30	1.7	18	<0.00002	33	3.7	9	0.00002
Extensin_2	20	11.0	2	0.00316	Protein kinase	30	1.7	18	<0.00002	33	14.0	2	0.01536
Cyclin_C	4	0.3	15	<0.00002	Protein kinase	30	1.7	18	<0.00002	26	0.3	76	<0.00002
Drf_FH1	11	4.3	3	0.00128	Protein kinase	30	1.7	18	<0.00002	19	5.5	3	0.01278
Cyclin	8	0.5	16	<0.00002	RIO1	11	0.3	39	<0.00002	19	0.3	59	<0.00002
Rrm	12	4.3	3	0.00032	Protein kinase	30	1.7	18	<0.00002	18	5.5	3	0.01692

The top 10 domain pairs observed in the interaction list are shown. As expected from interaction profiles (see text), cyclin and protein kinase domains are significantly associated, as are F-box and Skp domains. RIO1 is a recently described kinase domain [62] while the Extensin_2 domain is a proline-rich sequence. Drf_FH1 is the Diaphanous-related formin domain, a low-complexity 12-residue repeat found in proteins involved with cytoskeletal dynamics and the Rho-family GTPases [63], and the Rrm is an RNA-recognition motif. There are also additional associations between protein kinase domains and nucleic acid metabolism domains (see Additional data file 5). These data demonstrate the capacity of relatively small sets of proteins to generate high-confidence domain associations. A complete list of all domains and domain pairs found in the interaction data is in Additional data file 5.

## References

[B1] Fields S, Song O (1989). A novel genetic system to detect protein-protein interactions.. Nature.

[B2] Finley RL, Brent R (1994). Interaction mating reveals binary and ternary connections between *Drosophila* cell cycle regulators.. Proc Natl Acad Sci USA.

[B3] Hudson JR, Dawson EP, Rushing KL, Jackson CH, Lockshon D, Conover D, Lanciault C, Harris JR, Simmons SJ, Rothstein R, Fields S (1997). The complete set of predicted genes from *Saccharomyces cerevisiae *in a readily usable form.. Genome Res.

[B4] Uetz P, Giot L, Cagney G, Mansfield TA, Judson RS, Knight JR, Lockshon D, Narayan V, Srinivasan M, Pochart P (2000). A comprehensive analysis of protein-protein interactions in *Saccharomyces cerevisiae*.. Nature.

[B5] Li S, Armstrong CM, Bertin N, Ge H, Milstein S, Boxem M, Vidalain PO, Han JD, Chesneau A, Hao T (2004). A map of the interactome network of the metazoan *C. elegans*.. Science.

[B6] Giot L, Bader JS, Brouwer C, Chaudhuri A, Kuang B, Li Y, Hao YL, Ooi CE, Godwin B, Vitols E (2003). A protein interaction map of *Drosophila melanogaster*.. Science.

[B7] Rain JC, Selig L, De Reuse H, Battaglia V, Reverdy C, Simon S, Lenzen G, Petel F, Wojcik J, Schachter V (2001). The protein-protein interaction map of *Helicobacter pylori*.. Nature.

[B8] McCraith S, Holtzman T, Moss B, Fields S (2000). Genome-wide analysis of vaccinia virus protein-protein interactions.. Proc Natl Acad Sci USA.

[B9] Bartel PL, Roecklein JA, SenGupta D, Fields S (1996). A protein linkage map of *Escherichia coli *bacteriophage T7.. Nat Genet.

[B10] Ito T, Chiba T, Ozawa R, Yoshida M, Hattori M, Sakaki Y (2001). A comprehensive two-hybrid analysis to explore the yeast protein interactome.. Proc Natl Acad Sci USA.

[B11] Bader GD, Hogue CW (2002). Analyzing yeast protein-protein interaction data obtained from different sources.. Nat Biotechnol.

[B12] Deane CM, Salwinski L, Xenarios I, Eisenberg D (2002). Protein interactions: two methods for assessment of the reliability of high-throughput observations.. Mol Cell Proteomics.

[B13] von Mering C, Krause R, Snel B, Cornell M, Oliver SG, Fields S, Bork P (2002). Comparative assessment of large-scale data sets of protein-protein interactions.. Nature.

[B14] Jansen R, Yu H, Greenbaum D, Kluger Y, Krogan NJ, Chung S, Emili A, Snyder M, Greenblatt JF, Gerstein M (2003). A Bayesian networks approach for predicting protein-protein interactions from genomic data.. Science.

[B15] Fromont-Racine M, Mayes AE, Brunet-Simon A, Rain JC, Colley A, Dix I, Decourty L, Joly N, Ricard F, Beggs JD, Legrain P (2000). Genome-wide protein interaction screens reveal functional networks involving Sm-like proteins.. Yeast.

[B16] Schwikowski B, Uetz P, Fields S (2000). A network of protein-protein interactions in yeast.. Nat Biotechnol.

[B17] Gyuris J, Golemis E, Chertkov H, Brent R (1993). Cdi1, a human G1 and S phase protein phosphatase that associates with Cdk2.. Cell.

[B18] Finley RL, Zhang H, Zhong J, Stanyon CA (2002). Regulated expression of proteins in yeast using the *MAL61-62 *promoter and a mating scheme to increase dynamic range.. Gene.

[B19] Zhong J, Zhang H, Stanyon CA, Tromp G, Finley RL (2003). A strategy for constructing large protein interaction maps using the yeast two-hybrid system: regulated expression arrays and two-phase mating.. Genome Res.

[B20] Barabasi AL, Oltvai ZN (2004). Network biology: understanding the cell's functional organization.. Nat Rev Genet.

[B21] Jeong H, Mason SP, Barabasi AL, Oltvai ZN (2001). Lethality and centrality in protein networks.. Nature.

[B22] Watts DJ, Strogatz SH (1998). Collective dynamics of 'small-world' networks.. Nature.

[B23] Saito R, Suzuki H, Hayashizaki Y (2002). Interaction generality, a measurement to assess the reliability of a protein-protein interaction.. Nucleic Acids Res.

[B24] Goldberg DS, Roth FP (2003). Assessing experimentally derived interactions in a small world.. Proc Natl Acad Sci USA.

[B25] Bader JS, Chaudhuri A, Rothberg JM, Chant J (2004). Gaining confidence in high-throughput protein interaction networks.. Nat Biotechnol.

[B26] Ashburner M, Ball CA, Blake JA, Botstein D, Butler H, Cherry JM, Davis AP, Dolinski K, Dwight SS, Eppig JT (2000). Gene ontology: tool for the unification of biology. The Gene Ontology Consortium.. Nat Genet.

[B27] FlyBase Consortium (2003). The FlyBase database of the *Drosophila *genome projects and community literature.. Nucleic Acids Res.

[B28] Vazquez A, Flammini A, Maritan A, Vespignani A (2003). Global protein function prediction from protein-protein interaction networks.. Nat Biotechnol.

[B29] Hybrigenics web site. http://www.hybrigenics.com.

[B30] Yu H, Luscombe NM, Lu HX, Zhu X, Xia Y, Han JD, Bertin N, Chung S, Vidal M, Gerstein M (2004). Annotation transfer between genomes: protein-protein interologs and protein-DNA regulogs.. Genome Res.

[B31] Ge H, Liu Z, Church GM, Vidal M (2001). Correlation between transcriptome and interactome mapping data from *Saccharomyces cerevisiae*.. Nat Genet.

[B32] Tong AH, Lesage G, Bader GD, Ding H, Xu H, Xin X, Young J, Berriz GF, Brost RL, Chang M (2004). Global mapping of the yeast genetic interaction network.. Science.

[B33] Tewari M, Hu PJ, Ahn JS, Ayivi-Guedehoussou N, Vidalain PO, Li S, Milstein S, Armstrong CM, Boxem M, Butler MD (2004). Systematic interactome mapping and genetic perturbation analysis of a *C. elegans *TGF-beta signaling network.. Mol Cell.

[B34] Kolonin MG, Finley RL (2000). A role for cyclin J in the rapid nuclear division cycles of early *Drosophila *embryogenesis.. Dev Biol.

[B35] Lane ME, Sauer K, Wallace K, Jan YN, Lehner CF, Vaessin H (1996). Dacapo, a cyclin-dependent kinase inhibitor, stops cell proliferation during *Drosophila *development.. Cell.

[B36] Reiss DJ, Schwikowski B (2004). Predicting protein-peptide interactions via a network-based motif sampler.. Bioinformatics.

[B37] Jackson PK, Eldridge AG (2002). The SCF ubiquitin ligase: an extended look.. Mol Cell.

[B38] Samanta MP, Liang S (2003). Predicting protein functions from redundancies in large-scale protein interaction networks.. Proc Natl Acad Sci USA.

[B39] Rives AW, Galitski T (2003). Modular organization of cellular networks.. Proc Natl Acad Sci USA.

[B40] Spirin V, Mirny LA (2003). Protein complexes and functional modules in molecular networks.. Proc Natl Acad Sci USA.

[B41] King AD, Przulj N, Jurisica I (2004). Protein complex prediction via cost-based clustering.. Bioinformatics.

[B42] Bu D, Zhao Y, Cai L, Xue H, Zhu X, Lu H, Zhang J, Sun S, Ling L, Zhang N (2003). Topological structure analysis of the protein-protein interaction network in budding yeast.. Nucleic Acids Res.

[B43] Bader GD, Hogue CW (2003). An automated method for finding molecular complexes in large protein interaction networks.. BMC Bioinformatics.

[B44] Gagneur J, Krause R, Bouwmeester T, Casari G (2004). Modular decomposition of protein-protein interaction networks.. Genome Biol.

[B45] Ewing B, Green P (2000). Analysis of expressed sequence tags indicates 35,000 human genes.. Nat Genet.

[B46] Aparicio SA (2000). How to count ... human genes.. Nat Genet.

[B47] Welcome to the Finley Lab. http://proteome.wayne.edu/finlabindex.html.

[B48] Kolonin MG, Finley RL (1998). Targeting cyclin-dependent kinases in *Drosophila *with peptide aptamers.. Proc Natl Acad Sci USA.

[B49] Golemis EA, Serebriiskii I, Finley RL, Kolonin MG, Gyuris J, Brent R, Ausubel FM, Brent R, Kingston RE, Morre D, Seidman JG, Struhl K (1998). Interaction trap/two-hybrid system to identify interacting proteins.. Current Protocols in Molecular Biology.

[B50] FlyGrid. http://biodata.mshri.on.ca/fly_grid/servlet/SearchPage.

[B51] IntAct Interaction database. http://www.ebi.ac.uk/intact/index.html.

[B52] Hermjakob H, Montecchi-Palazzi L, Bader G, Wojcik J, Salwinski L, Ceol A, Moore S, Orchard S, Sarkans U, von Mering C (2004). The HUPO PSI's molecular interaction format - a community standard for the representation of protein interaction data.. Nat Biotechnol.

[B53] Koonin EV, Fedorova ND, Jackson JD, Jacobs AR, Krylov DM, Makarova KS, Mazumder R, Mekhedov SL, Nikolskaya AN, Rao BS (2004). A comprehensive evolutionary classification of proteins encoded in complete eukaryotic genomes.. Genome Biol.

[B54] NCBI Clusters of Orthologous Groups database. ftp://ftp.ncbi.nih.gov/pub/COG.

[B55] Ito T, Tashiro K, Muta S, Ozawa R, Chiba T, Nishizawa M, Yamamoto K, Kuhara S, Sakaki Y (2000). Toward a protein-protein interaction map of the budding yeast: a comprehensive system to examine two-hybrid interactions in all possible combinations between the yeast proteins.. Proc Natl Acad Sci USA.

[B56] Ho Y, Gruhler A, Heilbut A, Bader GD, Moore L, Adams SL, Millar A, Taylor P, Bennett K, Boutilier K (2002). Systematic identification of protein complexes in *Saccharomyces cerevisiae *by mass spectrometry.. Nature.

[B57] Gavin AC, Bosche M, Krause R, Grandi P, Marzioch M, Bauer A, Schultz J, Rick JM, Michon AM, Cruciat CM (2002). Functional organization of the yeast proteome by systematic analysis of protein complexes.. Nature.

[B58] Mewes HW, Amid C, Arnold R, Frishman D, Guldener U, Mannhaupt G, Munsterkotter M, Pagel P, Strack N, Stumpflen V (2004). MIPS: analysis and annotation of proteins from whole genomes.. Nucleic Acids Res.

[B59] CYGD: MIPS Comprehensive Yeast Genome Database. http://mips.gsf.de/proj/yeast/CYGD/interaction.

[B60] Flybase. http://flybase.net.

[B61] Celniker SE, Wheeler DA, Kronmiller B, Carlson JW, Halpern A, Patel S, Adams M, Champe M, Dugan SP, Frise E (2002). Finishing a whole-genome shotgun: release 3 of the *Drosophila melanogaster *euchromatic genome sequence.. Genome Biol.

[B62] Angermayr M, Bandlow W (2002). RIO1, an extraordinary novel protein kinase.. FEBS Lett.

[B63] Wallar BJ, Alberts AS (2003). The formins: active scaffolds that remodel the cytoskeleton.. Trends Cell Biol.

