# Supplementary material for: A Drosophila protein-interaction map centered on cell-cycle regulators
Source: Genome Biol. 2004 Nov 26;5(12):R96. doi: 10.1186/gb-2004-5-12-r96 (PMC545799; doi:10.1186/gb-2004-5-12-r96)
Supplement: Additional data file 1 — Supplementary Materials and methods [file gb-2004-5-12-r96-s1.rtf]

Supplementary Materials and Methods

Plasmids and strains

Plasmid maps and sequences can be found at proteome.wayne.edu.  The BD plasmid for expressing LexA-fused proteins from the MAL62 promoter, pHZ5-NRT, was previously described [18].  The AD vector used in generating the Drosophila AD array, pJZ4-NRT (also known as pJZ4-G), was derived from pJG4-5 [17], a 2µm TRP1 vector for expression of B42AD-fused proteins from the yeast GAL1 promoter.  The main differences are that pJZ4-NRT has a CYC1 terminator instead of an ADH1 terminator, and an F1 origin.  Construction of pJZ4-NRT proceeded in several steps. First, an adaptor was inserted into EcoRI/XhoI digested pJG4-5, to generate pRF4-5o. Next, a second adaptor, containing two additional rare restriction enzyme sites (Sbf1 and PmeI), was inserted into the multiple cloning site of pRF4-5o to generate pJF1.  This vector was designed to be used for making nested deletions using exonuclease III (J. Fonfara Lewis and R.L.F., unpublished). Starting from pJF1, two subclonings were performed to construct pJZ4. First, the CYC1 terminator (fragment 127 – 376) was amplified from pMyr (Stratagene) using primers JZ4 and JZ5 (JZ4 5'-AGA GAC TCG AGG GCG CGC CTA GGC CGG CCA TCA TGT AAT TAG TTA TGT CAC GC-3'; JZ5 5'-AGA GAG TCG ACG CAA ATT AAA GCC TTC GAG CG-3'). Primer JZ4 encoded two rare restriction enzyme sites, AscI and FseI.  The PCR product was digested with SalI and XhoI, and subcloned into pJF1 which had been digested with SalI and XhoI to remove its ADH1 terminator. The resulting plasmid was called pJZ4-1. Second, the F1 origin was amplified from pMyr (fragment 5052 - 5424) using primers JZ6 and JZ7 (JZ6 5'-AGA GAG TCG ACA ATT GTA AAC GTT AAT ATT TTG TTA AAA T-3'; JZ7 5'-AGA GAG TCG ACC CCG CTC CTT TCG CTT TCT TC-3'), and the PCR product was digested with SalI and subcloned into pJZ4-1 linearized by SalI. The resulting plasmid is called pJZ4.  Finally, a sequence, G4BD (TTG ACT GTA TCG GGC CTT AAG CCC GGG CCT TCC CTG CAG CCA AGC TAA TTC CGG) was inserted into pJZ4 linearized with EcoRI and XhoI by recombination subcloning in yeast. The fragment G4BD contained a 5' recombination tag (5RT1), followed by EcoRI and BamHI sites, and a 3' recombination tag (3RT1). The two recombination tags are homologous to the ends of the Drosophila ORFs amplified in Giot et al. [6].  The resulting plasmid was called pJZ4-NRT. 

Yeast array construction – additional details

Yeast transformation using the LiOAc method [47] with the following modifications to accommodate the large scale.  Competent yeast were prepared ahead of time and frozen at -80oC in 10% DMSO after adding ssDNA (final concentration 0.6 µg/µL) and linearized vector (final concentration 2.4 ng/µL). On the day of transformation, competent yeast were thawed and aliquoted into 96-well PCR plates at 10 µL per well. After adding 5 µL of PCR product and 30 µL of 40% PEG into each well, we placed the PCR plates into a thermo cycler for heat shock (42oC, 15 min). After adding 50 µL liquid medium containing 15% glycerol into each well, we plated 5 µL onto agar plates poured in 6-well Costar plates, and then placed the agar plates onto a rotating Nutator platform to distribute yeast unevenly on the agar.  The plates were incubated at 30oC for 3 days, then moved to room temperature.  Colonies were manually picked into 96-well plates.  For the AD array, one set was picked by combining, typically, 5 colonies per transformation into a single position of the array.  Two other versions of the array were also constructed, each using single colonies.  Three versions of the BD array were constructed, each using single colonies from the BD transformations.

Assessing the AD array
We estimated the frequency of inserts in the AD array by testing random plates by PCR.  We used yeast cells as template, and primers flanking the cloning site in pJZ4-NRT.  In one plate (plate ID 1229044), of 93 yeast clones tested, we determined the insert frequency for both the array generated with single colonies and the array generated with multiple (~5) colonies. Clones yielding no PCR product were excluded from the calculation. The insert frequency in the culture from multiple colonies was 89%, and among the 83 clones with an insert, 71 had a single insert, 3 had two different sized inserts, and 9 had a mix of insert and empty vector. The frequency of single inserts in the single-colony array was 98%. This result suggests that 89% - 98% of the clones in the AD array will have inserts. It also suggests that pooling ~5 single colonies does not increase the frequency of obtaining yeast clones with inserts; in other words, if a clone containing the inserted PCR product is not identified in the first colony, picking more yeast transformants generally will not help.  Moreover, pooling colonies increases the difficulty in identifying the insert responsible for the two-hybrid phenotype.

BD clones used in the screens

A set of 239 ORFs were selected from the Release 1 & 2 predicted D. melanogaster ORFs [63] (the Berkeley Drosophila Genome Project) based on the following criteria: genes annotated as cell cycle regulators, DNA replication/repair, apoptosis regulators, genes with sequence similarity to known cell cycle regulators and DNA repair proteins, and a number of additional S/T kinases (Supplemental Table 1). Of these, 154 were successfully subcloned into the BD vector, sequenced to verify insert identity and fidelity of ORF fusion with the vector, and subsequently used in the screens. (Supplemental Table 1).  The 154 different genes screened in this work were represented by 196 strains as indicated in Supplemental Table 1.  As previously described, clones from different putative transcripts of the same gene are indicated by the same gene identifier due to the difficulty in determining alternative transcripts [6].    Some of the 154 BDs were obtained from the BD array and some were subcloned separately, as detailed in the following paragraph.

BD strains corresponding to 183 ORFs were retrieved in triplicate from the three single colony BD arrays. Yeast DNA was extracted in 96-well format using a zymolyase/heat-lysis protocol to lyse the yeast [47] and silica-matrix/glass filter 96-well DNA binding plates (Promega, Whatman) to purify the DNA. Inserts were detected by PCR amplification with vector-specific primers. Insert-positive PCR reactions were purified by Sephadex filtration and sequenced. 105 BD strains, representing 88 different genes, that passed all these quality control tests, were used in the first set of two-phase pooled matings.  The 120 ORFs that failed to produce sequence-verified yeast strains by this approach were retrieved from the array of PCR products. Of these, 58 successfully re-amplified using primers homologous to the common recombination tag sequences. Additionally, some genes that failed to amplify were selected for amplification from three fly cDNA libraries [64], using gene-specific primers tailed with the recombination tags. This yielded an additional 17 PCR products. Finally, 8 ORFs were retrieved by PCR from the AD clones (diploid positives obtained screens with the first set of BD strains), for a total of 81 PCR products. 

All successfully amplified linears were re-amplified using primers homologous to the common tail sequences, with an additional 20 bases of 5' homology to the BD vector, and used in recombination cloning into pHZ5-NRT the same way the BD array was constructed. Yeast DNA was extracted as described above from pools of transformants, and plasmids were cloned through KC8 bacterial transformation [47]. Bacterial DNA of single clones was extracted by silica matrix technology, multiple plasmid preparations were tested for inserts by PCR with vector-specific primers, and ORF identities and junctions were sequence-verified. This resulted in a set of 71 plasmids, representing 69 genes, which were introduced into yeast strain Y309.  This set of BDs, presented in a single 96-well panel, were screened against 6 pools of AD strains from the sequence-verified set of ADs isolated with the first set of BDs, and individual one-on-one matings were performed to verify these interactions.

Two-hybrid screening – additional details

The two-phase pooled mating approach is very sensitive and capable of detecting weak interactions, interactions involving proteins that are toxic to yeast, and interactions with BD-fused proteins that activate transcription of the reporters on their own [19].  As with most assays, however, increases in sensitivity are likely to result in increases in the number of false positives detected.  To reduce the chances of including false positives in our dataset, we selected interactions for verification based on two generally accepted, though not rigorously proven ideas.  The first idea is that interactions are more likely to be false positives if they involve a protein with a large number of interactions.  The second idea is that stronger interactions are more likely to be biologically relevant; strengths can be roughly estimated from the level of two-hybrid reporter activity [65].  Thus, we selected a set of positives for verification based a combination of the number of interactions per BD or AD protein and the sum of the two-hybrid reporter activity scores (c_sum), which ranged from 1-8.  We re-tested all interactions with BDs having ≤ 25 interactions; with BDs having ≤ 100 interactions where the combined reporter scores were ≥ 6; and where the BD had ≤ 100 interactions and the AD had ≥ 2 interactions.

In addition to the pooled mating approach, we used the same set of 154 BD fusions to conduct library screening, essentially as previously described [4, 6].  We constructed the library by combining all ~13,000 AD yeast strains from the AD array (see Array Construction details).  Matings were conducted by combining 100 µL of saturated culture of BD strain with 100 µL of the AD Array library (diluted to OD600=3.5, or ~8x10^6^ colony forming units) into the same well of a 96-well filter plate (MSHV, Millipore).  Excess liquid was vacuumed out of the wells and the filter was placed onto YPD plates for 2 days at 30oC. Cells were washed with sterile water and then re-suspended in liquid diploid-selection media containing 2% galactose, 0.5% raffinose, and 0.5% maltose (GRM) to induce expression of the AD and BD fusions, respectively.  After incubation for 5 hours at 30oC, media was removed and replaced with GRM diploid selection media lacking leucine with 15% glycerol and frozen at –80oC.  To continue the screen, aliquots were thawed and plated onto GRM diploid selection media to determine plating efficiency and to the same media lacking leucine to select yeast expressing the LEU2 reporter.  Leu+ positive were picked and the Leu and lacZ phenotypes were tested in the presence of glucose and GRM to remove false positives in which the reporters are expressed independently of the AD or BD.  The remaining positives were then picked and the AD inserts were PCR amplified from the yeast using primers flanking the insertion site in pJZ4-NRT. The ORF identities were determined by 5' end sequencing to generate an "interaction sequence tag" or IST [66].  Some PCR products were determined to belong to the same restriction fragment class (RFC) as an IST by digestion with HaeIII; these were not sequenced.  The numbers of ISTs and RFCs for each interaction are shown in Supplemental Table 2.

The majority of interactions, 1641, were obtained from the pooled mating approach and 212 by the library approach; 39 of these interactions were found by both approaches, and involved 21 of the 44 BD genes active in both approaches. There were 95 genes for which interaction data was obtained by the pooled mating approach, and 59 genes in the case of the library screening approach. The average number of interactions was 18 per BD gene, with a range of 1 to 84, for the pooled mating data, while the library screening data had an average of only 4 interactions per active BD fusion.

Other interaction datasets

The LexA interactions detected in this study, along with the genetic interactions and reference interactions noted in Table 3 are listed in Supplemental Table 2; interlog interactions noted in that table are available by request.  The genetic interactions were downloaded from Flybase [59]  Reference interactions were determined by manual curation of peer-reviewed primary literature by searching PubMed [67] and Flybase for publications mentioning one of the BD proteins used in the LexA and Gal4 screens.  The lists of potential interlogs were generated as follows.  Eukaryotic clusters of ortholog groups (KOGs) [52], determined from 7 complete eukaryotic genomes, were downloaded on 1 August 2003 from NCBI [53]. This file defined 4852 clusters, each having proteins from at least three organisms.  Each cluster has a unique KOG ID.  Because the IDs used in this file were not Flybase Gene IDs (CG#), but GeneBank IDs, a program was created to automatically retrieve every GeneBank record associated with the KOGs, extract all the Flybase Gene IDs, and transform all fly IDs in into the corresponding CG numbers.  Yeast (S. cerevisiae) protein-protein interactions in the files "complex052102.tab" and "PPI_290403.tab", were downloaded from the MIPS [57] database [58]. The yeast gene names were transformed into ORF IDs to be consistent with KOGs file. In the Co-IP result ("complex052102.tab"), binary interactions are included between the bait protein and every protein that was co-precipitated, but not between the precipitated proteins (hub and spoke model) [55, 56].  Worm (C. elegans) protein interactions were obtained from Supplement Table 5 of Li et al. [5]. Worm protein names were extracted and transformed into the corresponding ORF IDs to be consistent with the Ids in the KOGs file.  The yeast and worm interaction lists were used to generate a list of pairing relationships between corresponding KOGs Ids.  These lists were then used to generate a list of potential Drosophila interlogs by pairing every Drosophila protein belonging to the first KOG with every Drosophila protein belonging to the second KOG, for each pair of KOGs.  Note that multiple Drosophila interactions can be generated from a single yeast or worm interaction. 

Data Enrichment Analyses

Analysis of AD, BD, and interaction lists for enrichment of single and paired functions according to the Gene Ontology (GO) database (Supplemental Tables 3 and 4), was accomplished by replacing genes in AD or BD lists with randomly selected genes from Release 3.1 of the Drosophila proteome predicted gene list [60].  A similar analysis was performed with PFAM domain annotations. To determine the average representation of each function or domain in each list, that could be expected due to chance, this replacement was repeated 50,000 times.  With each iteration, the actual number of representative genes or binary interactions was compared with the experimental number.  After 50,000 iterations, the number of occasions when the randomly generated data set had fewer representative genes than the experimental set was tallied, and the total was divided by 50,000 to generate an empirical P value (Pe(Exp>Rand)).  
The PFAM structure of each peptide in Drosophila release 3.1 was determined by the "HMMER" program [68], while the functional annotations were generated from the GO database as follows. Since any given annotation could have occurred at any distance from the base classification of "biological process" (BP), we created a list of all GO BP functions with all possible branch structures. Thus, a GO BP function placed 5 branch points from the base BP function would have 4 additional functions, super-ordinate in the GO hierarchy to the specific functional classification. This list was then used to infer classifications for any classified gene at specific distances from the BP base, or levels of classification. So, a gene with a "level 5" annotation would also have classifications at each super-ordinate level. We analyzed three levels of classification, starting at the second level or branch point below the BP base and including the third and fourth levels of the hierarchy. The fourth level of the GO hierarchy includes the functional annotation of "cell cycle".
