# Supplementary material for: A Drosophila protein-interaction map centered on cell-cycle regulators
Source: Genome Biol. 2004 Nov 26;5(12):R96. doi: 10.1186/gb-2004-5-12-r96 (PMC545799; doi:10.1186/gb-2004-5-12-r96)
Supplement: Additional data file 6 — Supplementary Table 5: P-values for overlap among datasetsa nd Supplementary Table 6: Interactions from the LexA and Gal4 screens that successfully used the same BD bait proteins [file gb-2004-5-12-r96-s6.pdf]

**Supplementary Table 5**. P values for overlap among datasets

| | Interactions | Overlap with LexA map (N=1,814) | Overlap with Gal4 map (N=20,439) |
|---|---|---|---|
| Yeast interlogs (hub/spoke) | 67,238 | 24 (1.32%; P<0.0001) | 251 (1.23%; P<0.0001) |
| Yeast interlogs (matrix)* | 244,202 | 25 (1.46%; P<0.0001) | 294 (1.44%; P<0.0001) |
| Worm interlogs | 37,863 | 3 (0.17%; P<0.1436) | 61 (0.30%; P<0.0001) |
| Drosophila genetic | 2,751 | 4 (0.22%; P<0.0024) | 22 (0.11%; P<0.0001) |
| Reference set | 47 | 8 (0.44%P<0.0001) | 6 (.03%; P<0.0001) |
| Ref set (common BD) | 20 | 3 (0.17%; P<0.0001) | 2 (.01%; P<0.0001) |

Data from Table 3. P values indicate the chances of finding the number of overlapping interactions with a random set of interactions. The random sets were generated by replacing the AD proteins in each of the maps with a random set or proteins, keeping the BD proteins, the number of interactions per BD protein and AD protein, and the topologies of the maps the same (see Methods).

**Supplementary Table 6.** Interactions from the LexA and Gal4 screens that successfully used the same BD bait proteins.

| | Interactions | Overlap with LexA map (N=859) | Overlap with Gal4 map (N=243) | Overlap in common |
|---|---|---|---|---|
| Yeast interlogs (hub/spoke) | 67,238 | 19 (2.21%) | 6 (2.47%) | 4 |
| Worm interlogs | 37,863 | 3 (0.35%) | 1 (0.41%) | 0 |
| Drosophila genetic | 2,751 | 1 (0.12%) | 4 (1.65%) | 0 |
| Ref set (common BD) | 20 | 3 (0.35%) | 2 (.82%) | 0 |

Of the 154 BDs used in both screens (Supplementary Table 1), 14 had interactions only in the Gal4 screens, 60 had interactions only in the LexA screens, and 46 were used successfully to detect interactions in both the LexA and Gal4 screens. With these 46 BDs, the LexA screens detected 859 interactions and the Gal4 screens detected 243. The table shows the overlap among these interactions and the 3 datasets as described in Table 2 and Methods.
