# Supplementary figures and images for: A Drosophila protein-interaction map centered on cell-cycle regulators
Source: Genome Biol. 2004 Nov 26;5(12):R96. doi: 10.1186/gb-2004-5-12-r96 (PMC545799; doi:10.1186/gb-2004-5-12-r96)

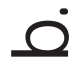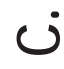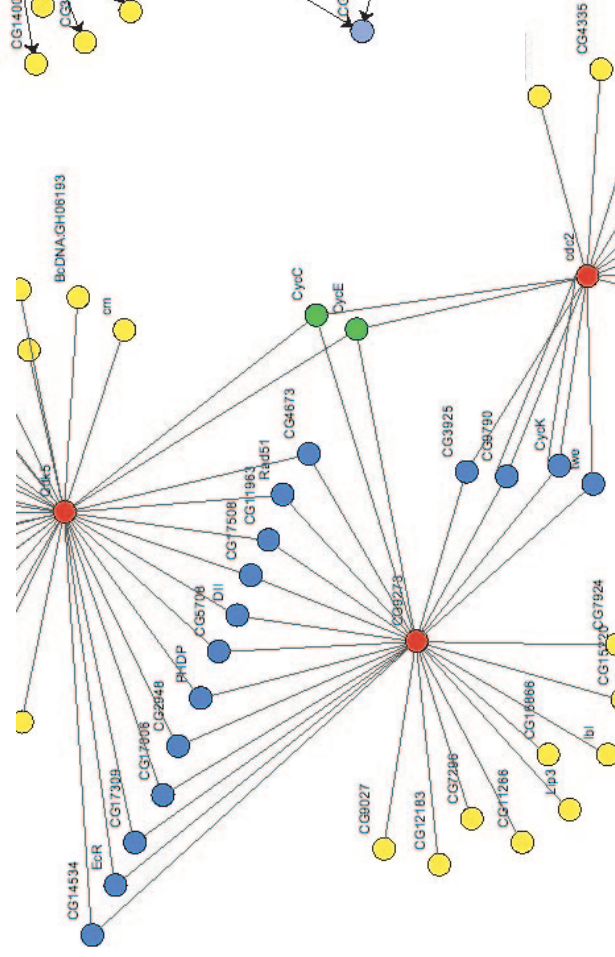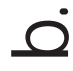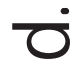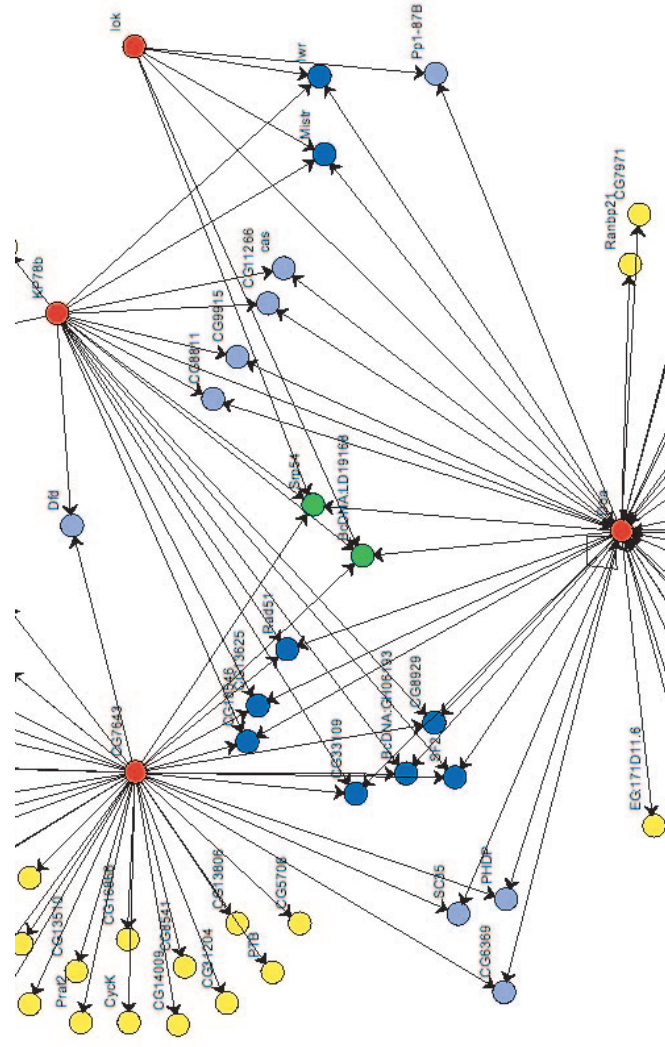

Supplement: Additional data file 7 — Supplementary Figure 1: Interaction maps of other clusters [file gb-2004-5-12-r96-s7.pdf]

BD Proteins

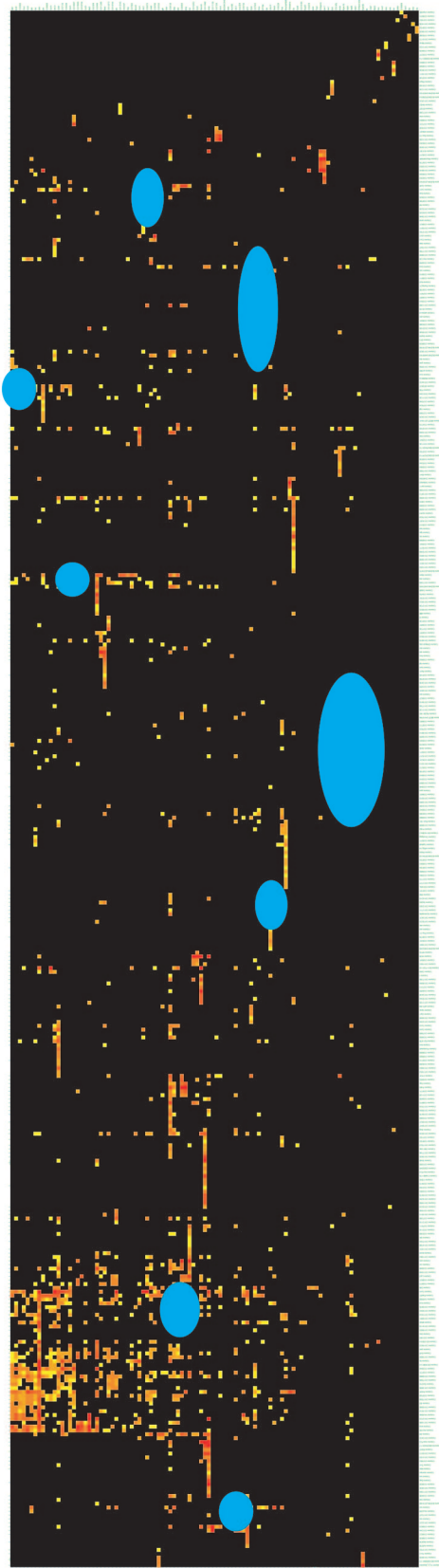

AD Proteins

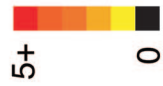

Supplement: Additional data file 8 — Supplementary Figure 2: Proteins clustered by interaction profile [file gb-2004-5-12-r96-s8.pdf]
