# Supplementary material for: A Drosophila protein-interaction map centered on cell-cycle regulators
Source: Genome Biol. 2004 Nov 26;5(12):R96. doi: 10.1186/gb-2004-5-12-r96 (PMC545799; doi:10.1186/gb-2004-5-12-r96)
Supplement: Additional data file 9 — The legends to Supplementary Figures 1 and 2 [file gb-2004-5-12-r96-s9.doc]

Additional data file 9.

Legends for Additional data file 7 and 8.

**Supplementary Figure 1.** Interaction maps of other clusters. Several clusters of interacting proteins derived from Fig. 3. The red colored proteins in each cluster have similar interaction profiles. **(a)** Proteins that interact with Par-1 and DNAprim (red), which include 6 proteins that interact with both (green). **(b)** Most of the proteins that interact with Dap, Plu, CG8455, Png, Rux, and CG8128 (red). **(c)** Most of the proteins that interact with Cdk5, Cdc2, and CG9293 (red), including 16 proteins (blue) that interact with at least two of these and 2 proteins that interact with all three (green). **(d)** Most of the proteins that interact with KP78b, CG7643, Doa, and Lok (red), including 2 that interact with all four red proteins (green), 9 that interact with at least three of the red proteins (dark blue), and 9 that interact with at least two of the red proteins (light blue). High resolution versions of these networks with live links to gene information can be drawn using a program available at proteome.wayne.edu [50].

**Supplementary Figure 2.** Proteins clustered by interaction profile. Full size version of the interaction profile shown in Fig. 3. AD-fused genes are on the x axis with gene names listed along the bottom of the figure. BD-fused genes are on the y axis with genes names listed on the right of the figure. The matrix shows whether or not the AD and BD genes interact: a black square indicates no interaction; a colored square indicates an interaction between the AD and BD genes that intersect with that square. The color of the square indicates the level of two-hybrid reporter gene activity, with yellow as the lowest and red as the highest. The genes are ordered based on the similarity of their interaction profiles. The figure is an output file from the Genespring hierarchical clustering program (Methods).
